# The Effect of Electrode Materials on the Fusion Rate in Multi-State Fusion Reactors

**DOI:** 10.3390/ma18163734

**Published:** 2025-08-09

**Authors:** Mahmoud Bakr, Tom Wallace-Smith, Keisuke Mukai, Edward Martin, Owen Leighton Thomas, Han-Ying Liu, Dali Lemon-Morgan, Erin Holland, Talmon Firestone, Thomas B. Scott

**Affiliations:** 1 School of Physics, University of Bristol, Bristol BS8 1TL, UK; edward.martin@bristol.ac.uk (E.M.); t.b.scott@bristol.ac.uk (T.B.S.); 2 Astral Systems LTD, Bristol BS10 7SB, UK; tomws@astralsystems.com (T.W.-S.); owen@astralsystems.com (O.L.T.); han-ying@astralsystems.com (H.-Y.L.); dali@astralsystems.com (D.L.-M.); erin@astralsystems.com (E.H.); talmon@astralsystems.com (T.F.); 3 Physics Department, Assiut University, Assiut 71516, Egypt; 4 National Institute for Fusion Science, 322-6 Oroshi-cho, Toki 509-5292, Gifu, Japan; mukai.keisuke@nifs.ac.jp; 5 Institute of Advanced Energy, Kyoto University, Uji 611-0011, Kyoto, Japan

**Keywords:** multi-state fusion, neutron production rate, surface fusion, neutron sources, lattice confinement fusion

## Abstract

This study assesses how different anode materials influence neutron production rates (NPRs) in multi-state fusion (MSF) reactors, with a particular focus on the effects of deuterium (D) pre-loading on the anode surface. Three types of mesh anodes were assessed: stainless steel (SS), zirconium (Zr), and D pre-loaded zirconium (ZrD). MSF operates using two electrodes to confine ions to various fusion reactions, including D-D and D-T. The reactor features a negatively biased central cathode and a grounded anode within a vacuum vessel. Neutrons and protons are produced through the application of high voltage (tens of kV) and current (tens of mA) on the system to spark the plasma and start the fusion. Assessments at voltages up to 50 kV and currents up to 30 mA showed that Zr mesh anodes produced higher NPRs than SS ones, reaching 1.912 at 30 kV. This increased performance is attributed to surface fusion processes occurring in the anode. These processes were further modified by the deuterium pre-loading in the ZrD anode, as compared to SS and Zr with 1.832 at 30 kV. The findings suggest that material properties and deuterium pre-loading play significant roles in optimizing the efficiency of MSF reactors and the NPR. Future research may explore the long-term stability and durability of these anode materials under continuous operation conditions to fully harness their potential in fusion energy applications.

## 1. Introduction

Inertial electrostatic confinement (IEC) fusion is a well-established nuclear fusion technique known for trapping light atoms like deuterium (D) and tritium (T) within a potential well to generate subatomic particles [1,2,3,4]. The IEC system typically features a concentric cathode connected to a negative bias and is surrounded by a grounded anode that serves as a vacuum vessel. Its straightforward structure, ease of handling, and cost-effectiveness have made it a focal point of research and application in the last three decades. The operational principle of the IEC is as follows: The IEC’s chamber is evacuated and then backfilled with fuel gas at several pascals. A current of tens of milliamperes (mA) is passed through the cathode, causing heating and generating electrons for gas ionization [5,6]. Alternative methods for gas ionization in the IEC include employing an external electron source or an external ionizing source. These methods provide flexibility and offer potential advantages in specific scenarios [7]. Applying tens of kilovolts (kV) between the electrodes establishes a potential well in the chamber, sparking a discharge and initiating plasma within the system. The ionized gas is accelerated toward the center of the cathode, leading to fusion reactions between the accelerated ions within the electrodes. These fusion reactions produce both atomic and subatomic particles, including neutrons, protons, tritons, helium-3, and helium-4, see Equation (1) as follows:

(1) D++D+→He23+n2.45 MeV50%→T13+P3.03 MeV50%D++T+→He24+n14.03 MeV

Particles (particularly neutrons) play a crucial role in the applications of IEC systems, and they are generated through various fusion paths, such as beam–beam reactions, beam–background gas reactions, and beam–surface reactions [1]. The contribution of each reaction to the total neutron production rate (NPR) depends on a range of factors, including operating conditions like gas pressure, applied voltage, grid current, and electrode properties such as geometry, shape, and material. Studies have extensively explored the impact of operating conditions on NPR, revealing key relationships. NPR is directly proportional to the grid current at constant applied voltage and gas pressure, and it increases exponentially with applied voltage while decreasing with gas pressure at a fixed grid current [2]. Additionally, research has addressed the influence of cathode shape and material on NPR, particularly under moderate operating conditions and low cathode temperature [3]. Two electrodes, SS and titanium, were investigated to clarify the effect of cathode materials on NPR in two different experimental setups [1,4]. Cathode material with high affinity to hydrogen isotopes has been found to enhance surface fusion and, consequently, the NPR [4,5,7,8]. Analysis of previous studies indicated that materials with high deuterium affinity produced more neutrons due to surface fusion or fusion occurring within the lattice of the material, referred to as lattice confinement fusion (LCF) [9], during system operation under certain temperature level. Fusion events primarily take place in material rather than in gas or plasma, shifting the emphasis to enhancing material-based fusion instead of gas-phase or plasma-phase fusion. In the IEC system, which focuses on both gas phase and electrode phase fusion, this approach will be termed multi-state fusion systems (MSF). LCF occurs on electrode surfaces due to D buildup during system operation [9].

The current study examines the effect of the anode material and the D pre-loaded electrodes on NPR. More precisely, the goal is to assess the influence of anode material and the D deposition on the anode materials on the NPR of the MSF reactor. Stainless steel alloy 316 (SS-316) and Zr meshes were made as spherical mesh anodes for the MSF system. D-D Performance comparisons based on applied voltage, grid current, NPR with SS, Zr, and ZrD are collected and discussed. In Section 2, the experimental setup and conditions are elaborated upon. Section 3 presents the results and discussion of the experiment, followed by conclusions and suggestions for future work.

To explain the enhancement of the NPR as a function of the current by changing the anode material (specifically the mesh material), one will begin by discussing the parameters affecting the NPRs generated from the MSF device. The total NPR in the system can be considered as a combination of three main fusion processes within the system volume, as discussed in the introduction section: beam–beam, beam–background, and beam-electrode surface interactions [1]. The contribution of each type varies based on several parameters, including system configurations, dimensions, electrode materials, and operational conditions. The total neutrons generated from the system can be theoretically calculated using Equation (2) as follows [7]:

(2) $$NPR≅\frac{I\sigma }{e}\left(N_{gas}+N_{surf}\right)$$

(3) $$N_{gas}≅\frac{2\eta rP}{K_{B}T(1-\eta^{2})}\;\mathrm{and}\;N_{surf}≅\left(N_{emb}+N_{ads}\right)$$

where I (mA) is the grid current comprising electron flow rate and positive ion flow rate; e (C) is the electron charge; σ (m^−2^) is the fusion cross-section; N_gas_ and N_surf_ are the target gas density and target surface density, respectively, where N_gas_ involves fusion reactions between energetic ions and background gas targets that generate neutrons and N_surf_ refers to surface fusion reactions on electrodes that generate neutrons and includes adsorbed N_ads_ and embedded N_emb_ target densities on cathode and anode surfaces; r (cm) is the cathode radius; k_B_ is Boltzmann’s constant; P (Pa) and T (K) are the gas pressure and temperature, respectively; and h is the cathode transparency. The cathode used in the current experiment has a radius of 2.5 cm in 97.5% transparency. The gas temperature entered the MSF system is kept at 20 °C, and the gas pressure during the system operation is ranging between 1 Pa and 3 Pa.

## 2. Experimental Setup and Conditions

### 2.1. Apparatus

**Figure 1:**
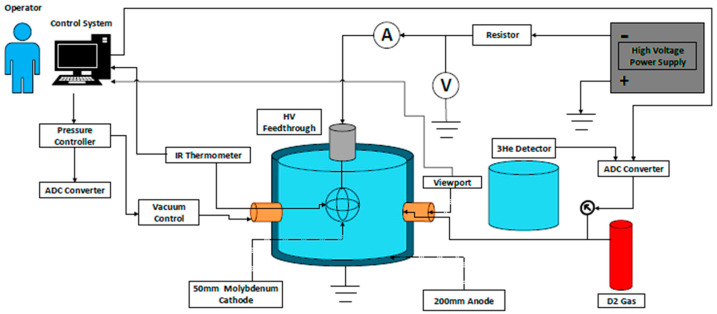
Schematic drawing to the IEC facility used in the test.

The MSF system consists of a cylindrical chamber fabricated from stainless steel alloy 304 (SS-316), serving as a vacuum vessel connected to a ground bias, as shown in Figure 1. The cylindrical shape was chosen for this test due to its simple structure and cost-effectiveness. The chamber has an inner diameter and height of 20 cm, with a wall thickness of 0.5 cm. The cathode is constructed from six Molybdenum (Mo) rings, each with a diameter of 5 cm, a width of 0.3 cm, and a thickness of 0.05 cm. The cathode is connected to a feedthrough system via a stainless-steel stalk, which in turn connects to a high-voltage cable through a 50 cm ceramic connector. The MSF system is powered by an 8 kW Glassman power supply operating in constant current mode, providing up to 100 kV and 80 mA, as shown in the sketch of Figure 1.

The neutrons are generated from the system, with an energy of 2.45 MeV, and are detected using a fast neutron detection system calibrated with a ^252^Cf source prior to use. To establish the calibration factor (neutrons per count), a ^252^Cf source with an intensity of 1.39 × 10^3^ ns^−1^ is positioned at the center of the vacuum chamber for 20 h. Neutron counts are conducted using a ^3^He detector (dimensions: 2.5 cm × 30 cm, 4 atm) that operates through D (d, n) ^3^He reactions. The detector, surrounded by a lead sheet and a 10 cm high-density polyethylene moderator, is situated 40 cm from the MSF system’s center. Signals from the detector are collected using an ORTEC package. The package included a pre-amplifier module (model 142C), powered by a 1.5 kV bias supply module (model 478). Pre-amplified signals are sent to an amplifier module (model 570), and the counts are processed through a ratemeter module (model 661) to generate an analog output. This analog output is converted to a digital signal via an analog-to-digital converter and monitored live on the control system, providing real-time neutron yield data. Additionally, the amplified signal is analyzed using a multichannel analyzer (model MCA8000D, AMETEK, Middleboro, MA, USA) and corresponding interfacing software (DPPMCA display and acquisition software DP5), as sketched in Figure 1. This setup is used to determine the gross area of neutron counts over a specific period (neutron/count). The resulting count rate (number of counts per second) is then multiplied by the calibration factor to estimate the NPR.

**Figure 2:**
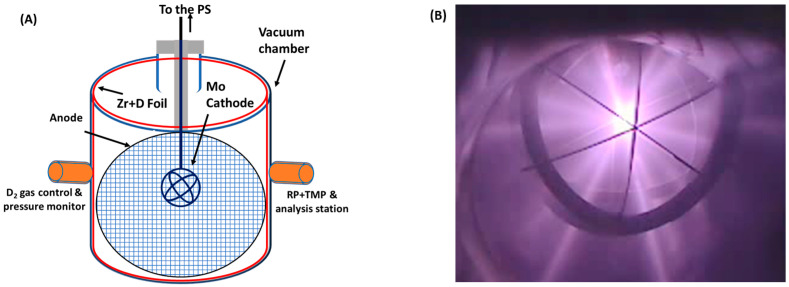
(**A**) A schematic representation of the MSF chamber, featuring a Mo cathode, Zr mesh (anode), and cylindrical Zr foil (depicted by the red line). (**B**) The plasma during the system operation at 30 mA current and 30 kV voltage.

The cathode temperature is measured using an IR thermometer through a side window of the vacuum vessel. Additionally, three thermocouples are attached at various positions on the vessel’s surface to measure the outer surface temperature of the wall. The cathode temperature and the outer surface temperatures of the chamber are recorded as a function of the applied voltage and current. Subsequently, the average temperature is estimated according to the location and time stamp. D gas is introduced into the chamber through a controlled leaking valve, with precise flow-rate control of ±0.01 Pa, achieved through an interface program managed by an analog–digital converter (ADC). The chamber is evacuated and maintained at a base pressure of ~10^−5^ Pa using a combination of a rotary pump and a turbo-molecular pump. The gas pressure, typically from 1.0 Pa to 5.5 Pa, is continually monitored during the system operation. Control and monitoring of the system parameters, as well as data acquisition, are facilitated by LabVIEW (V-2021) software, interfacing with ADCs. This software enables precise regulation of set parameters and real-time monitoring of output values during experiments [3]. Figure 2A presents the schematic diagram of the MSF chamber, illustrating the components including the Mo cathode, Zr mesh (depicted in blue), and cylindrical Zr foil (indicated by the red line). Figure 2B displays a photograph of the cathode operating under discharge conditions at 30 kV and 30 mA.

### 2.2. Sample Preparations

Two Zirconium foils of purity >99.99%, with dimensions of 20 cm width, 65 cm length, and a thickness of 0.01 cm, were used for control and D-preloaded meshes. The dimensions were chosen to cover the inner curved surface of the vacuum vessel with holes made for airflow at the location of each opposing viewport. In addition, a wire mesh consisted of 5 m of wire of Zr with a purity of >99.99% and a diameter of 0.1 cm. The D enrichment was performed via electrolysis using a 10 × 30 mm Pt mesh electrode in 1000 mL of DD oxide solution consisting of one mol of potassium hydroxide. The electrolysis was performed in a spherical reaction flask situated in a 3 L water bath to provide additional thermal mass, limiting solution evaporation, and maintaining a temperature of 70 °C during deuterolysis. A ‘TENMA 72-13360 300 W 60V15 A Wide Range Programmable DC Power Supply’ was used to apply 5 V and 3 A across the platinum electrode, Zr foil, and wire samples for four hours. The distance between the platinum electrode and Zr samples was approximately 20 mm in both cases. SIMS shows clear D content with depth, whilst Scanning electron microscopy (SEM) images show darker areas of Zr deuteride appearing after the deuterolysis. The samples were also visibly darkened after the deuterolysis process [9,10]. The estimated concentration of D on the Zr wire or foil was estimated to be 0.3 to 1 mg kg^−1^. A SS mesh without electrolysis and it has the same wire thickness and length was prepared in this study for comparison.

## 3. Results and Discussion

The system was evaluated under varying voltage, pressure, current, mesh material, and foil conditions. Grid currents ranged from 5 mA to 30 mA and voltages from 10 kV to 50 kV, using different meshes (SS, Zr, and ZrD) and foils (Zr and ZrD). Experiments were conducted with fixed Mo cathode material. In the first set, SS, Zr, and ZrD meshes surrounded by Zr foil were used as anode materials. In the second set, SS and ZrD meshes surrounded by ZrD foil were experimented. Each voltage/current and NPR scan is performed three times for consistency and performance tracking. The results plotted in next figures use the average NPR values and standard deviation as the error bar. In the next subsection an example of the experimental results with and without D pre-loading effect on the mesh (anode), along with the other properties investigations.

### 3.1. Paschen’s Curve

**Figure 3:**
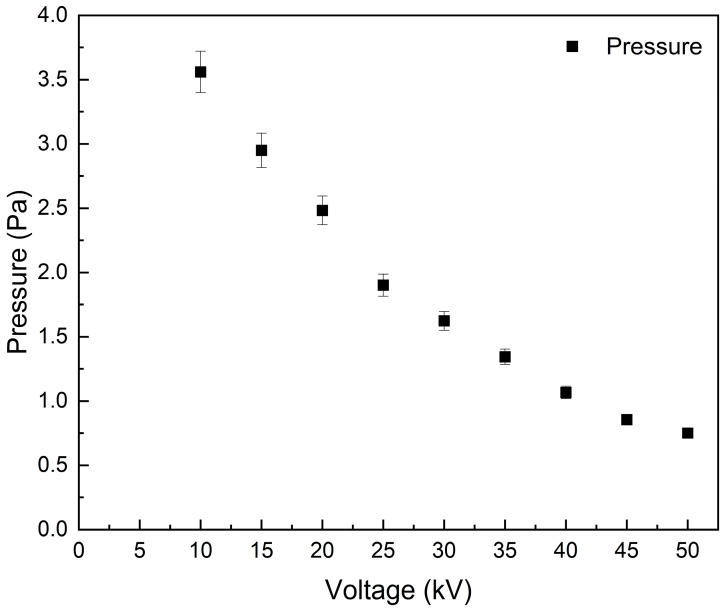
The breakdown voltage as a function of the gas pressure for the MSF system with a Mo cathode and SS mesh at 5 mA grid current.

The Paschen’s curve, pressure–voltage relationship (P-V) curve, or the breakdown voltages as a function of the gas pressure of a plasma system, is a unique relationship between the applied voltage and the gas pressure at a constant grid current in the vacuum vessel [4]. The Paschen’s curve changes based on the vacuum system configurations, gas type to spark the plasma, electrode materials, and operation conditions in the system. The P-V curve for the current configuration is measured at a constant current of 5 mA, with voltages ranging from 10 kV to 50 kV for the different meshes and foils. The system was configured with a Mo cathode and an SS mesh as the anode. The resulting P-V curve, shown in Figure 3, reveals that the breakdown voltage is influenced by gas pressure when the current remains steady at 5 mA. The observed results align with the trend predicted by Paschen’s law in different systems. It is worth noting that the breakdown pressure was slightly higher with Zr, ZrD meshes, or foils. This can be attributed to Zr’s greater affinity for D compared to SS, which results in more gas pressure needed to spark plasma with the same voltage. This observation agrees with previous experimental results for P-V under similar conditions [5,10,11]. In all experiments, the same Mo cathode is used at a constant current of 5 mA. The thermionic emission rate remained unchanged, resulting in similar ionization rate. Variations in breakdown voltage were due to changes in gas pressure from D adsorption on the Zr mesh or foil.

### 3.2. Influence of Electrode Temperatures on Fusion Rate Measurements

**Figure 4:**
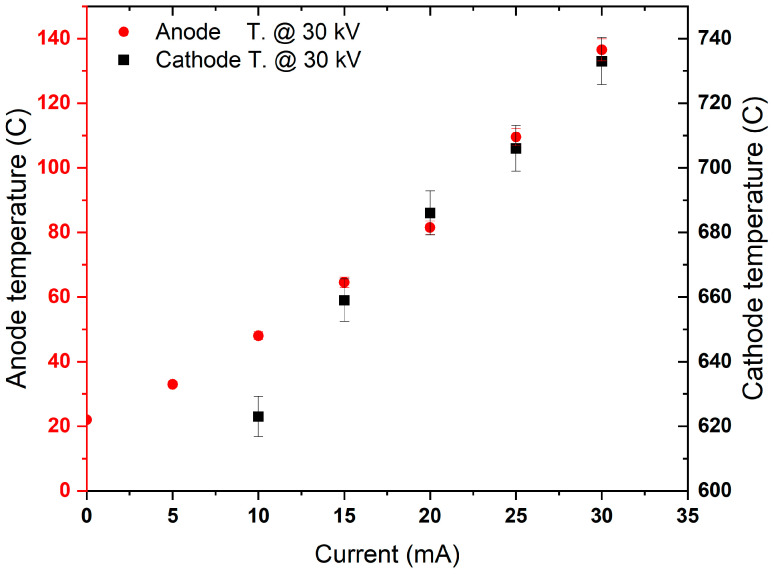
The temperature profile of the cathode and anode surface as a function of grid current at a constant voltage of 30 kV.

The vacuum chamber lacked a water-cooling jacket, limiting operational time and conditions due to anode surface heating, which reduced system performance and NPR stability. The main heat sources were radiation from the cathode and electron jets escaping through cathode apertures. A new chamber, with a 40 cm diameter, height, and a 2 cm water jacket, was fabricated to cool the anode’s inner surface, allowing for a higher grid current of 100 mA and an applied voltage of 120 kV. Data with high NPR variation due to heating effect (above 20% variations) were excluded from this study; therefore, only 30 kV cathode voltage and 30 mA grid current were considered. Figure 4 displays the measured temperatures of the cathode and anode surfaces (averaged across various positions of the anode) without Zr foil on the inner chamber surface. Temperatures were recorded as a function of grid current at a constant 30 kV voltage. The data indicates that increasing grid current raises anode temperature due to radiation from the cathode and electron jets from the cathode openings. The linear relationship between the cathode and anode temperature is consistent with other systems measurements [12]. It is noteworthy that at elevated current and temperature levels, the NPR stability exhibited significant fluctuations during each data collection.

### 3.3. Effect of the Input Power on the Fusion Rate

**Figure 5:**
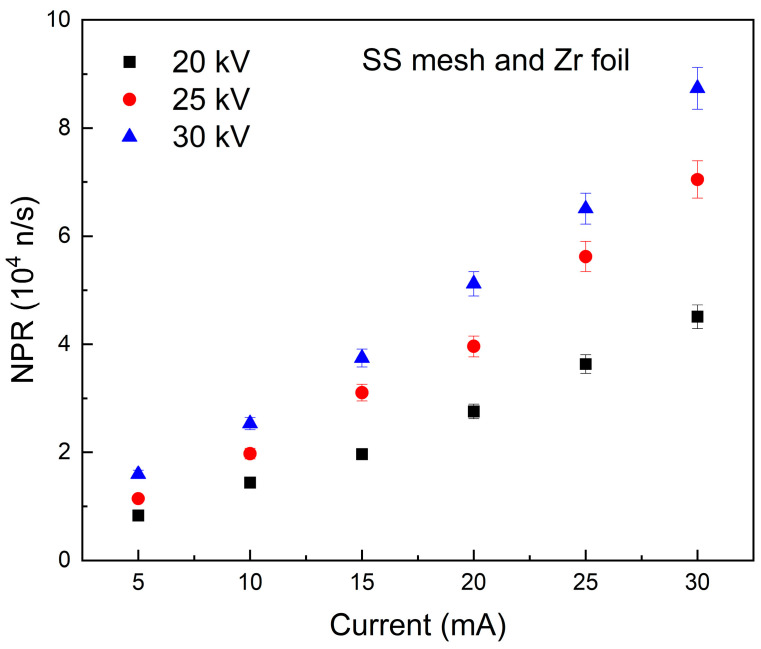
The NPR of the SS mesh and Zr foil (chamber wall) at varying applied voltages.

**Figure 6:**
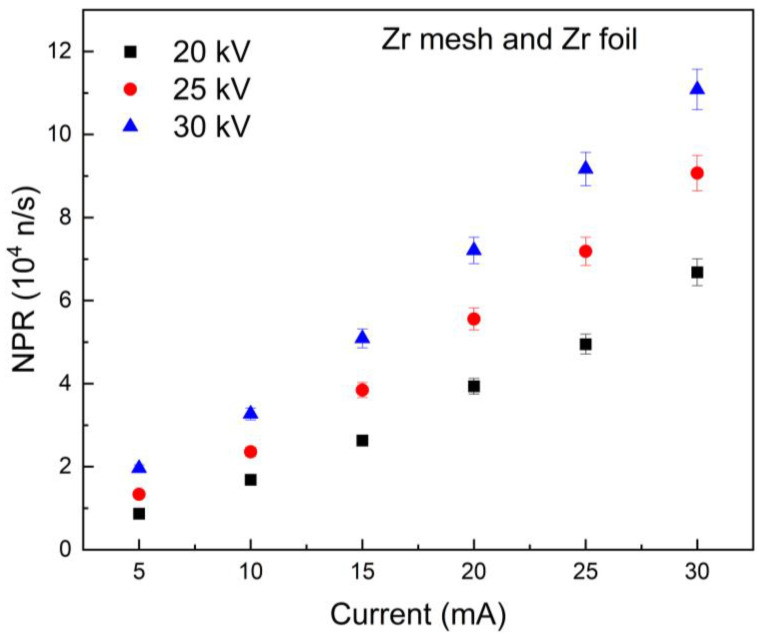
The NPR of Zr mesh and Zr foil (chamber wall) as a function of current at various voltages.

Figure 5 and Figure 6 illustrate the relationship between NPR and grid current at 20 kV, 25 kV, and 30 kV for the MSF system, with SS and Zr meshes as respective anodes and Zr foil surrounding the inner wall of the chamber. The results indicate a linear relationship between NPR and the current within the applied voltage range. This tendency aligns with observations from other chambers featuring different configurations [13]. Improvement in NPR as a function of the applied voltage at a constant current is also expected; however, it is higher than in the same setting without the Zr foil (directly on the chamber surface). This may be attributed to the Zr foil on the surface of the chamber, which has a high affinity for adsorbing D, thereby increasing the likelihood of surface fusion, and consequently enhancing the NPR.

The enhancement of NPR with applied voltages from 20 kV to 25 kV, 25 kV to 30 kV, and 20 kV to 30 kV was quantified from Figure 5 and Figure 6. For SS and Zr meshes with Zr foil, the enhancements were 1.439 ± 0.096 and 1.476 ± 0.061, respectively, for 25/20 kV. For 30/25 kV, the NPR ratios were 1.262 ± 0.081 for SS mesh and 1.329 ± 0.088 for Zr mesh. For 30/20 kV, the values were 1.861 ± 0.073 for SS mesh and 1.912 ± 0.096 for Zr mesh. The Zr mesh demonstrated a higher improvement compared to the SS mesh due to its greater affinity for deuterium. This allows for increased D adsorption on the wire surface and enhances the probability of fusion events, leading to an improved total neutron output from the system. It should be noted that the contribution of the Zr foil to the total neutron output remains consistent in both the SS and Zr mesh experiments.

### 3.4. Effect of the Anode Material on the Fusion Rate

**Figure 7:**
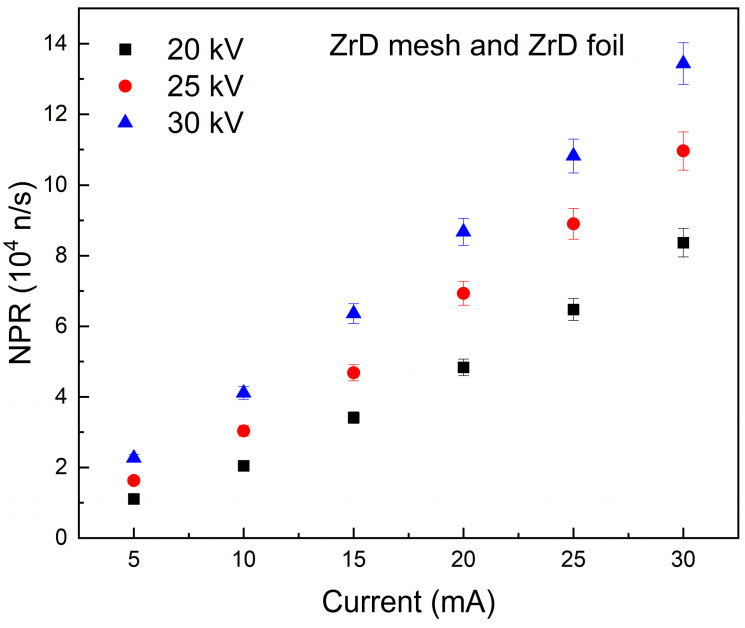
The NPR of ZrD mesh and ZrD foil as a function of current at various voltages.

Figure 7 illustrates the NPR as a function of grid current at varying applied voltages from the IEC system that utilizes both ZrD mesh and ZrD foil with the same Mo cathode. The evaluated enhancement of the NPR for the applied voltages ranging from 20 kV to 25 kV, 25 kV to 30 kV, and 20 kV to 30 kV was 1.409 ± 0.068, 1.299 ± 0.076, and 1.832 ± 0.178, respectively. The ZrD mesh exhibited superior NPR improvement owing to the presence of D in the mesh prior to operation, which increased the fusion probability on the surface of both the mesh and the chamber wall, consequently enhancing the NPR. Neutrals that do not fuse with D on the mesh can still fuse with D on the chamber wall, ZrD foil, which offers more surface area.

**Figure 8:**
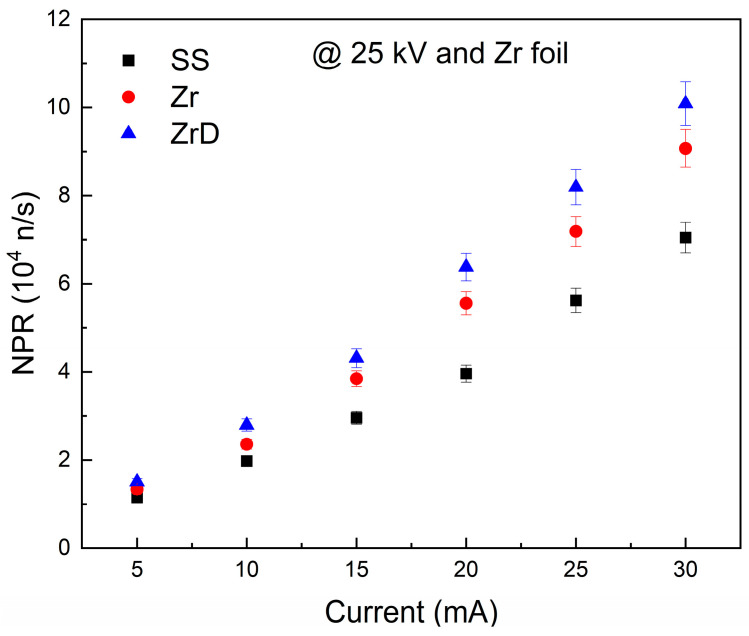
The NPR as a function of the grid current at 25 kV for different meshes and Zr foil.

Figure 8 compares the NPR for various meshes SS, Zr, and ZrD using the same Mo cathode materials and Zr chamber wall (no D pre-loading) at 25 kV. The NPR’s trend aligns with expectations based on grid current. Calculated NPR ratios for Zr/SS, ZrD/SS, and ZrD/Zr are 1.272 ± 0.085, 1.447 ± 0.097, and 1.138 ± 0.026, respectively. D affinity and preloaded D in the mesh affect NPR, despite the low density of D (0.3 mg kg^−1^ to 1 mg kg^−1^). Theoretically, higher NPR correlates with increased D concentration in the mesh. It can be seen from the figures and the comparison values that the system with ZrD mesh generated higher NPR compared to Zr and SS meshes in the system, indicating that the enriched Zr introduced more fusion events and hence NPR.

To understand the results, let us examine how neutrons are generated in the MSF system. Neutron production depends on three main fusion probabilities. Beam–beam fusion is controlled by input voltage and current in the MSF system and represented by the term (I_c_/e) in Equation (2). Increasing current, simply the number of electrons, raises the number of ionized gas particles, linearly increasing fusion events as seen in Equation (2) and the tendency depicted in Figure 6, Figure 7 and Figure 8. Higher voltage increases the ions’ kinetic energy, boosting the fusion cross-section and neutron output, as shown in Equation (2). This study compared grid current and applied voltage, finding that they do not affect the total number of fusion events or NPR. In summary, beam–beam fusion does not account for variations in neutron outputs in the current investigation. Beam–background fusion in the MSF system is controlled by gas pressure (N_gas_ in Equation (2)). Higher gas pressure increases the number of ions if enough electrons are available for ionization through the grid current. More ionized gas may increase fusion events and neutron yield. However, at constant grid current, higher gas pressure lowers grid voltage, reducing fusion cross-section and hence neutron output—a trade-off relationship. Since the cathode properties and the gas temperature are fixed in the current study, the fusion events due to the N_gas_ contributions remains constant because of the constant values of the gas pressure, transparency, temperature, and the applied voltage in the comparison, so the N_gas_ cannot explain the NPR differences in the above figures.

Beam–surface fusion events in the MSF are influenced by two distinct components: the fusion events occurring due to adsorbed D ions (N_ads_) and embedded ions (N_emb_) in both the cathode and anode. The positive ions are exclusively accelerated towards the center, cathode rings and the system center, by the acceleration field, subsequently becoming adsorbed or embedded into the cathode surface based on their kinetic energy, the higher the energy, the deeper they penetrate the cathode. Notably, as the cathode temperature rises, there is an increased likelihood of the adsorbed D ions being released back into the plasma, thus reducing the fusion events attributable to N_ads_ from the cathode. The probability of shallow adsorbed ions being released into the plasma escalates with increasing cathode temperature. In the current study, during NPR measurements, the cathode material, and operational conditions (applied voltage, current, and gas pressure) remained nearly consistent, indicating that the contribution of N_surf_ from the cathode was uniform and could not account for the variations in neutron output across different experimental setups. The sole modification in this experiment was the anode materials specifically, SS, Zr, and ZrD meshes. Beam–surface fusion on the anode is like that on the cathode, with notable differences. Since the anode is grounded, positive ions avoid the mesh, while electrons and neutrals from charge exchange processes interact with it. Neutrals (D*, $D_{2}^{*}$ ), formed from ion charge loss, bombard the system components including the cathode, mesh, and chamber wall. The charge exchange processes are described in Equation (4):

(4) $$D^{+*}+D\to D^{*}+D^{+},\;D_{2}^{+*}+D_{2}\to D_{2}^{*}+D_{2}^{+}$$

Neutrals are deposited or embedded in the electrodes based on their energy during bombardment, facilitating fusion events. The fusion rate varies with material properties and surface temperature; materials with high D adsorption affinity show higher fusion rates, while higher temperatures reduce them. Neutral contributions from the cathode and chamber walls remain constant due to identical materials and conditions throughout the experiment. However, the mesh’s contribution will vary because the mesh material will influence the neutral adsorption, desorption, and embedding process.

Understanding the affinity of the mesh materials to retain D on their surface requires knowledge of the stopping range and energy deposition of electrons and neutrals in the material surface. When electrons hit a material, they lose energy by interacting with atoms, converting most kinetic energy into thermal energy, which heats the material surface. The electron range helps evaluate deep penetration effects, such as heating properties and disruption of the material to the D. The stopping range (R) is the material thickness where the transmission curve drops to zero.

**Figure 9:**
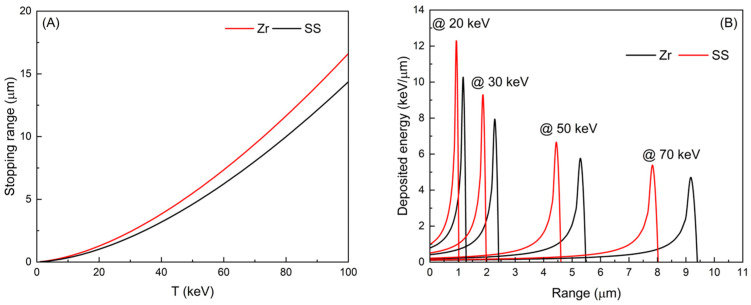
The electron stopping range as a function of electron energy (**A**). Additionally, the relationship between deposited energy and range for electrons at different energies (20 keV up to 70 keV) within Zr and SS meshes (**B**).

At low energies, R is determined by linearly extrapolating the number energy curve for a given absorber thickness. For high atomic number absorbers, extrapolation uses the tangent at the steepest point [14,15]. The range (R) of electrons with energy from 0.3 keV to 30 MeV in absorbers (atomic number 6–92) can be described by a single semi-empirical Equation as follows [14]:

(5) $$R=\frac{a_{1}}{\rho }\left[\frac{\ln(1+a_{2}\tau )}{a_{2}}-\frac{a_{3}\tau }{1+a_{4}\tau^{a_{5}}}\right],\;a_{1}=\frac{2.335A}{Z^{1.209}},\;a_{2}=1.78\times 10^{-4}Z,\;a_{3}=0.9891-\left(3.01\times 10^{-4}Z\right),\;a_{4}=1.468-\left(1.180\times 10^{-2}Z\right),\;a_{5}=\frac{1.232}{Z^{0.109}}$$

where ρ (kgm^−3^) represents the absorber density, specifically the cathode material density. τ denotes the incident kinetic energy in units of electron rest energy. The parameters a_i_ (i = 1, to 5) are simple functions of the atomic number Z and atomic weight A. For Zr the values of A and Z are used directly and then plotted in Figure 9A. While for SS different strategy is used because it is a compound of varied materials. For mixtures or compounds, replace Z and A with their effective values, Z_eff_ and A_eff_, respectively.

(6) $$Z_{\mathrm{eff}}=\sum_{i}f_{i}Z_{i},\;A_{\mathrm{eff}}=\frac{Z_{\mathrm{eff}}}{(Z/A{)}_{\mathrm{eff}}},\;(Z/A{)}_{\mathrm{eff}}=\sum_{i}\frac{f_{i}Z_{i}}{A_{i}}$$

The fraction by weight of the constituent element with Z_i_ and A_i_, the effective atomic number, and the weight for SS-316 were calculated, used in Equation (6), and then used to calculate the stopping range of SS plotted in Figure 9A. The heat deposited by electrons inside the absorber materials, which increase mesh temperature during the system operation, is defined as the energy lost ΔE (eV) over an infinitesimal material thickness ΔR (μm), given by ΔE/ΔR [14]. The energy deposited into the Zr and SS meshes is plotted against the stopping range at 20 keV, 30 keV, 50 keV, and 70 keV electron energy in Figure 9B. The figure illustrates that the stopping range of electrons in Zr mesh is anticipated to be longer compared to SS mesh, which can be attributed to the material densities of 6.49 g/cm^3^ for Zr and 8 g/cm^3^ for SS. Additionally, the distribution of deposited energy on the surface of the mesh at four different electron energies suggests that electrons follow Bragg’s curve, meaning they gradually lose energy through straggling within the mesh lattice and deposit significant amounts of energy just before coming to rest, as observed in the figure. Electrons deposit higher energy near the surface of the SS mesh compared to the Zr mesh under identical electron energy, resulting in more heat on the SS mesh surface compared to Zr.

Furthermore, the projected stopping range and energy deposition of neutrals with energies of 50 keV and 100 keV were calculated using SRIM when embedded in Zr and SS materials [14]. The projected stopping ranges were 311 nm and 264 nm for 50 keV, and 573 nm and 463 nm for Zr and SS, respectively, closely aligning with the electron stopping range trend, considering differences in mass and charge. The deposited energy, which will convert to heat and potentially cause deposition of other D atoms if no fusion event occurs, is 502 eV/μm and 777 eV/μm at 50 keV, and 317 eV/μm and 509 eV/μm at 100 keV for Zr and SS, respectively. These results affirm the electron trend that energy deposited before the neutral comes to a stop in Zr is less than in SS, implying stronger heat generation in SS compared to Zr. Additionally, the deeper energy deposition in Zr compared to SS reduces surface heating of the Zr mesh, consequently lowering the probability of adsorbed D deposition from the Zr mesh compared to the SS mesh. The discussion suggests that Zr mesh is likely to retain more D than SS mesh under the same conditions in the MSF system. This increases the number of D being stacked in Zr compared to SS, which increase the chances for surface fusion, leading to higher NPR in Zr mesh compared to SS one. If D atoms are pre-attached to the Zr mesh (e.g., ZrD mesh), the D concentration would be even higher, further increasing the probability of fusion events and hence the NPR, as shown in Figure 8.

More research is necessary to understand the effects of anode and cathode materials on NPR [16,17,18,19,20]. Future studies should increase D deposition on the mesh to enhance fusion probability and NPR. Planned simulations and experiments will investigate this further. Measuring mesh temperature within the MSF system can clarify energy transfers during operation. Using techniques like SEM or Transmission Electron Microscope (TEM) to analyze material structure before and after exposure to plasma will provide insights. Potential anode materials such as Molybdenum, Titanium, Aluminum, and Tungsten will be evaluated for their impact on fusion rates in the MSF system.

## 4. Conclusions and Future Work

This study investigated the influence of the anode (mesh) material and the D pre-loading effect on the neutron production rate in a multi-state fusion device. Three different mesh anodes—SS, Zr, and ZrD—were fabricated and examined at various applied voltages up to 30 kV and grid currents up to 30 mA, while other system parameters, including cathode properties and system configurations, were kept constant. The experimental results indicated that higher neutron production rates are achievable using Zr mesh anodes compared to SS mesh anodes. This performance enhancement is attributed to surface fusion or lattice confinement fusion occurring between neutrals and D atoms deposited on the mesh surface in Zr is higher than in SS. Additionally, it was observed that pre-loaded D on the ZrD mesh material further increased the neutron production rate compared to the Zr mesh, due to the concentration of D on the mesh surface increasing the probability of fusion events with neutrals generated from charge exchange in the MSF system. Assessments at voltages up to 50 kV and currents up to 30 mA showed that Zr mesh anodes produced higher NPRs than SS ones, reaching 1.912 at 30 kV. This increased performance is attributed to surface fusion processes occurring in the anode. These processes were further modified by the deuterium pre-loading in the ZrD anode, as compared to SS and Zr with 1.832 at 30 kV. Future work aims to comprehensively understand the effect of electrode materials on neutron production rates for the MSF, including pre-loading different anode materials with D, varying D concentrations on the anode surface, and exploring different pre-loading techniques. Furthermore, extending the operating power regime will help better understand the surface fusion effect on NPRs, several types of fusion, and optimal conditions for maintaining plasma at higher input power levels to maximize neutron output from the MSF system.

## Data Availability

The original contributions presented in this study are included in the article. Further inquiries can be directed to the corresponding authors.
