# Correction: Bakr et al. The Effect of Electrode Materials on the Fusion Rate in Multi-State Fusion Reactors. *Materials* 2025, *18*, 3734

**DOI:** 10.3390/ma19010137

**Published:** 2025-12-31

**Authors:** Mahmoud Bakr, Tom Wallace-Smith, Keisuke Mukai, Edward Martin, Owen Leighton Thomas, Han-Ying Liu, Dali Lemon-Morgan, Erin Holland, Talmon Firestone, Thomas B. Scott

**Affiliations:** 1 School of Physics, University of Bristol, Bristol BS8 1TL, UK; 2 Astral Systems LTD, Bristol BS10 7SB, UK; 3 Physics Department, Assiut University, Assiut 71516, Egypt; 4 National Institute for Fusion Science, 322-6 Oroshi-cho, Toki 509-5292, Gifu, Japan; 5 Institute of Advanced Energy, Kyoto University, Uji 611-0011, Kyoto, Japan

In the original publication [1], there was a mistake in Figure 2B. The corrected Figure 2B appears below. The authors state that the scientific conclusions are unaffected. This correction was approved by the Academic Editor. The original publication has also been updated.

## Figures and Tables

**Figure 2:**
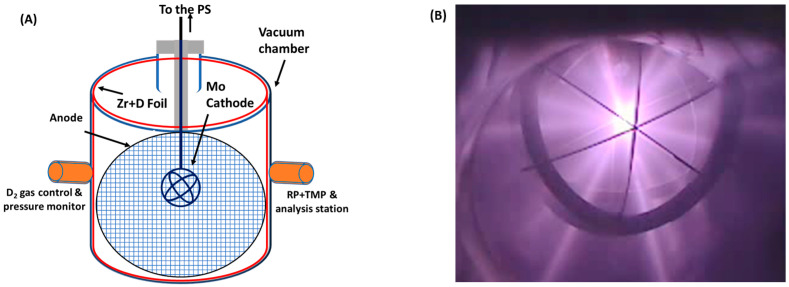
(**A**) A schematic representation of the MSF chamber, featuring a Mo cathode, Zr mesh (anode), and cylindrical Zr foil (depicted by the red line). (**B**) The plasma during the system operation at 30 mA current and 30 kV voltage.
